# HH-MAPPO: A Hierarchical Reinforcement Learning Framework for Dynamic-Scale Target–Attacker–Defender Games

**DOI:** 10.3390/e28070793

**Published:** 2026-07-13

**Authors:** Junhui Huang, Yan Guo, Xiliang Chen, Jianyu Wei, Jiawei Yi, Xinliang Chen, Lifeng Chen

**Affiliations:** 1 School of Communication Engineering, Army Engineering University of PLA, Nanjing 210000, China; 18351960676@163.com (J.H.);; 2 School of Command and Control Engineering, Army Engineering University of PLA, Nanjing 210000, China

**Keywords:** multi-agent, system, target–attacker–defender game, hierarchical reinforcement learning, behavioral diversity, role-aware embedding, energy efficiency

## Abstract

The Target–Attacker–Defender (TAD) pursuit–evasion game is a core challenge in multi-agent cooperative control, yet real-world settings involving dynamic team scaling and strict energy constraints remain largely unaddressed. When scalable shared-parameter policies are adopted to cope with the varying number of agents, severe policy homogeneity emerges, preventing effective division of labor. This paper proposes a Hierarchical Heterogeneous Multi-Agent Proximal Policy Optimization (HH-MAPPO) framework to resolve these challenges. Both levels employ actor–critic networks with Role-Aware Embedding (RAE). In this mechanism, each agent is assigned a unique, learnable role embedding derived from its identity. These embeddings serve as conditioning inputs to the shared policy network, enabling it to generate differentiated behaviors and effectively mitigating policy homogeneity. The upper-level policy determines the number of defenders to deploy and assigns interception targets, while the lower-level policy handles continuous control of each defender and the ground moving target (GMT). This hierarchy resolves dynamic observation spaces via a target-matching mechanism, where each defender’s observation includes only its own state and its assigned attacker’s state, keeping observation dimension constant. Experiments in a 3D TAD simulation with continuous attacker arrivals and energy-constrained defenders show the following: (1) HH-MAPPO achieves superior interception performance compared to baseline methods in both symmetric and asymmetric scenarios; (2) ablation studies confirm RAE increases policy diversity, raising Sequence-Based Action Dissimilarity (SBAD) by 15.5%; and (3) Pareto analysis demonstrates a superior performance–energy trade-off, maintaining about 70% interception rate even under an extreme energy cap (E = 30).

## 1. Introduction

Multi-agent collaboration, as a core research direction in interdisciplinary fields such as artificial intelligence and automation, addresses complex tasks through cooperation among intelligent agents [1,2,3,4,5,6]. Examples include large-scale inspection and security patrols, emergency rescue and target search, as well as autonomous cooperative defense. Among these, autonomous cooperative defense involves the typical Target–Attacker–Defender (TAD) three-party pursuit–evasion game [7,8,9,10], which is regarded as a highly challenging topic in the field of multi-agent cooperative control due to its significant dynamics and adversarial nature. This game involves three core roles: the attacker attempts to capture the target, the defender is responsible for intercepting the attacker to protect the target, and the target actively maneuvers to evade threats.

Traditional TAD game research primarily relies on differential game theory [11,12,13] and geometric analysis methods [14,15]. These works have established a solid theoretical foundation under simplified settings, such as two-dimensional planes, single entities per role, and no resource constraints [16,17,18,19,20]. Although recent studies have attempted to extend these to three-dimensional space or multi-agent scenarios [21,22,23], the model-driven nature of these methods makes it difficult to cope with the curse of dimensionality and modeling challenges posed by dynamic changes, resource constraints, and strong heterogeneity.

With the rapid advancement of computing power, artificial intelligence algorithms have been widely used in various fields [24,25,26]. Data-driven deep reinforcement learning methods have opened new avenues for complex game-theoretic decision-making due to their strong environmental interaction capabilities and scenario adaptability. In particular, existing research has largely focused on scenarios with communication constraints [27]. Specifically, in [28], a hierarchical multi-agent deep reinforcement learning architecture for 3D multi-agent TAD games under irregular underwater terrain and low-frequency communication constraints is proposed. In [29], the orbital maneuvering game of satellites with only pulsed propulsion capabilities is addressed. It is modeled as an optimization problem involving pulse magnitude, type, and mission objectives, and deep reinforcement learning is employed for its solution. In [30], underwater adversarial scenarios with communication and perception constraints are addressed, and a multi-agent deep reinforcement learning framework that indirectly shapes the attacker’s reward function to force retreat is proposed. However, these studies do not adequately address the core practical requirement of dynamic resource scheduling for both offensive and defensive sides during sustained confrontation. To tackle dynamic scale scheduling, in [31], a scalable MADDPG algorithm supporting dynamic adjustment of the number of unmanned surface vehicles during training is proposed; in [32], a scalable deep reinforcement learning framework for distributed penetration strategies under partial observability is designed. Nevertheless, most of these studies focus on scenarios with homogeneous agents. They fail to address heterogeneous multi-agent collaboration, particularly lacking consideration for the energy constraints of defense units and the active decision-making role of the moving target.

Therefore, this paper is dedicated to studying a TAD game scenario that integrates the aforementioned multiple complexities. Specifically, the scenario takes place in a three-dimensional closed bounded environment. The defensive side is strictly energy-constrained and faces continuously reinforcing attacker swarms. The ground-based moving target, which has autonomous decision-making and maneuvering capabilities, dynamically generates defense units based on the global situation to achieve efficient cooperative interception.

This scenario gives rise to two core and interrelated algorithmic challenges. First is the fundamental contradiction between scalability and policy differentiation brought about by dynamic scales. Using Actor–Critic networks with shared parameters is an inevitable choice for handling variable-scale teams [31,32,33], but this can lead to the policy homogeneity problem [34,35], where all agents exhibit identical behaviors, making it difficult to form effective divisions of labor in complex tasks.

To collaboratively address the aforementioned challenges, this paper proposes a novel architecture that integrates role representation learning with hierarchical task decomposition. This architecture is explicitly divided into two layers. The upper decision layer is responsible for determining the number of defenders to generate and for assigning interception targets. These decisions are based on the global situation, energy constraints, and attacker coverage principles. The lower control layer handles motion control: each defender optimizes efficient interception trajectories according to its assigned task, while the GMT observes the attacker’s state and generates evasive trajectories. Through structured decomposition and personalized representation, this architecture synergistically addresses the challenges of policy homogeneity and cooperative control in dynamic multi-agent scenarios.

The contributions of this study can be summarized as follows:

1. We propose an asymmetric hierarchical policy architecture for dynamic-scale TAD games. In this design, the GMT learns to make energy-aware decisions on how many defenders to deploy, while each defender learns which attacker to intercept. This asymmetric assignment of strategic roles to heterogeneous agents enables end-to-end joint optimization of resource scheduling and task assignment, which goes beyond fixed rule-based methods.
2. We introduce a dual-stream conditioned role-aware embedding (RAE) architecture that mitigates policy homogeneity in shared-parameter networks. The RAE first modulates each agent’s observation via its role embedding, and it then extracts task-oriented and role-specific features through separate processing streams before additive fusion. This structured mechanism produces complementary and mutually predictable behaviors essential for stable multi-agent coordination, rather than the unstructured divergence caused by naive independent layers.
3. Extensive experiments in a 3D TAD simulation with continuous attacker arrivals and strict energy constraints demonstrate that the proposed framework consistently outperforms existing baselines in both interception rate and energy efficiency. Ablation studies further reveal a key insight: effective teamwork depends on structured differentiation grounded in shared representations, rather than the mere magnitude of action dissimilarity.

## 2. Problem Formulation and Environment Modeling

### 2.1. Problem Formulation

**Figure 1:**
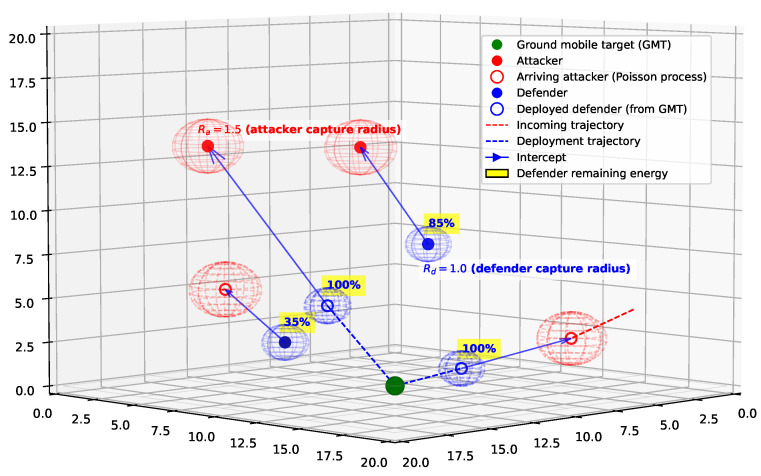
TAD confrontation scenario.

The adversarial scenario in this study involves three agent types: defenders, attackers, and ground mobile target (GMT) (Figure 1). Specifically, defenders and attackers are unmanned aerial vehicles (UAVs), and the GMT is an unmanned ground vehicle (UGV). Let the defender set be $D=\{d_{1},d_{2},\ldots ,d_{N}\}$, the attacker set be $A=\{a_{1},a_{2},\ldots ,a_{M}\}$, and the GMT be *C*. During the mission, the number of defenders *N* is dynamically adjusted by the reinforcement learning policy, while the number of new attackers arriving at each time step, denoted by *M*, is independently sampled from a Poisson distribution with mean λ (i.e., M∼Poisson(λ)) and placed at random positions on the boundary.

The motion of the Defender and GMT follows a second-order integrator model. As described in [36], energy consumption exhibits a clear coupling relationship with the required thrust, while thrust serves as the primary driving force for acceleration. Therefore, we can directly employ acceleration as the control input to construct the dynamic equations and energy consumption equations [37]. Specifically, the dynamic equation for the *i*-th defender can be expressed as follows:

(1) $$\begin{array}{cc} v_{d_{i}}^{t+1} & =v_{d_{i}}^{t}+a_{d_{i}}^{t}\cdot \Delta t\cdot \mu_{d},\\ p_{d_{i}}^{t+1} & =p_{d_{i}}^{t}+v_{d_{i}}^{t+1}\cdot \Delta t+\omega , \end{array}$$

where $v={\left[v_{x},v_{y},v_{z}\right]}^{T}\in R^{3}$ and $p={\left[x,y,z\right]}^{T}\in R^{3}$ denote velocity and position vectors, $a={\left[a_{x},a_{y},a_{z}\right]}^{T}\in R^{3}$ is the acceleration control input, Δt is the time step, $\mu_{d}$ is the propulsion efficiency coefficient of the defender, and ω∼N(0,0.05) models environmental disturbances.

Following the established relationship between quadcopter thrust *F* and the square of rotor speed $\sum w_{k}^{2}$ (i.e., $F=c_{F}\sum w_{k}^{2}$) as identified in [36], and considering that thrust is the primary force producing acceleration (a∝F), we model the energy consumption per step as a quadratic function of the acceleration magnitude:

(2) $$\Delta E_{d_{i}}=\frac{1}{2\chi_{d}}{∥a_{d_{i}}^{t}∥}^{2},\;\chi_{d}\in \left(0,1\right).$$

Here, $\chi_{d}$ represents the energy utilization efficiency of defenders, a parameter analogous to the inverse of the identified thrust coefficient $c_{F}$ in [36], encompassing propulsion system inefficiencies. When the residual energy $E_{d_{i}}$ of defender $d_{i}$ is less than the minimum operating energy $E_{d}^{\min}$ (i.e., $E_{d_{i}}<E_{d}^{\min}$), the defender enters an energy-saving mode where only deceleration is allowed:

(3) $$a_{d_{i}}^{t}=-\rho_{d}\cdot v_{d_{i}}^{t},$$

where $\rho_{d}$ is the deceleration coefficient controlling the rate of speed reduction.

The GMT moves in a two-dimensional plane (z=0), and its dynamics equation can be expressed as follows:

(4) $$\begin{array}{cc} v_{C}^{t+1} & =v_{C}^{t}+a_{C}^{t}\cdot \Delta t\cdot \mu_{C},\\ p_{C}^{t+1} & =p_{C}^{t}+v_{C}^{t+1}\cdot \Delta t+\omega , \end{array}$$

where $\mu_{C}$ is the propulsion efficiency coefficient of the GMT. Although ground vehicle resistance is more complex, we adopt a similar quadratic energy model for control consistency and to reflect increased energy cost during aggressive maneuvers. Its energy consumption is as follows:

(5) $$\Delta E_{C}=\frac{k}{\chi_{C}}{∥a_{C}^{t}∥}^{2},$$

where k=0.6 is a ground-specific coefficient and $\chi_{C}$ represents the energy utilization efficiency of GMT. When the residual energy $E_{C}$ of the GMT is less than the minimum operating energy $E_{C}^{\min}$ (i.e., $E_{C}<E_{C}^{\min}$), the GMT enters energy-saving mode, which only allows deceleration:

(6) $$a_{C}^{t}=-\rho_{C}\cdot v_{C}^{t}.$$

Attackers employ the same dynamic model as defenders. Notably, their behavior is not constrained by energy limitations. The dynamic equation for the *j*-th attacker is as follows:

(7) $$\begin{array}{cc} v_{a_{j}}^{t+1} & =v_{a_{j}}^{t}+a_{a_{j}}^{t}\cdot \Delta t,\\ p_{a_{j}}^{t+1} & =p_{a_{j}}^{t}+v_{a_{j}}^{t+1}\cdot \Delta t. \end{array}$$

This paper assumes the defender holds superiority in both velocity and acceleration, i.e., $|v_{d}^{max}\left|>\right|v_{a}^{max}\left|>\right|v_{C}^{max}|$ and $|a_{d}^{max}\left|>\right|a_{a}^{max}\left|>\right|a_{C}^{max}|$. The attacker and defender each possess capture radii $R_{A}$ and $R_{D}$ [17]. The game concludes when all attackers are captured by the defender, or when any attacker successfully captures the GMT. The core parameters of all agents are summarized in Table 1.

The optimization objective of this study is to achieve a Pareto optimal balance between interception rate and energy consumption, meaning to maximize the interception rate under a given energy constraint.

### 2.2. Environment Modeling

The complex TAD game, defined by dynamic reinforcements, energy constraints, and real-time GMT coordination, is fundamentally a multi-agent sequential decision-making problem with partial observability and heterogeneous agent objectives. Therefore, we formulate this problem as a Partially Observable Stochastic Game (POSG). This formalization is motivated by three key reasons: First, the requirement for defenders to make independent yet coordinated decisions based on local observations aligns with the decentralized execution principle inherent in POSG. Second, each unit’s inherently limited knowledge of teammates’ precise status and opponents’ full intentions satisfies the partially observable assumption. Finally, since each defender may have distinct role-specific objectives while contributing to a common team goal, POSG’s allowance for individual reward functions enables more precise credit assignment and role-aware learning.

The POSG is formally defined by the tuple 〈I,S,A,P,Ω,O,R,γ〉. The components are specified as follows:

(1) Agents (*I*): The set of learning agents comprises the defender *D* and the GMT *C*, i.e., $I=D\cup \left\{C\right\}=\left\{d_{1},\ldots ,d_{N},C\right\}$. The number of defenders *N* is dynamically adjusted by the GMT’s policy. The attackers include both learning and non-learning agents. The behavior of the non-learning agents is governed by an artificial potential field [38] (see [39] for details). The learning agents adopt the same algorithm as the defenders.

(2) State Space (*S*): The global state $s^{t}\in S$ at time *t* encapsulates all entities:

(8) $$\begin{array}{c} s^{t}=\left({\left\{s_{d_{i}}^{t}\right\}}_{i=1}^{N},\;{\left\{s_{a_{j}}^{t}\right\}}_{j=1}^{M},\;s_{C}^{t}\right), \end{array}$$

where $s_{d_{i}}^{t}=\left[p_{d_{i}}^{t},v_{d_{i}}^{t},E_{d_{i}}^{t}\right]$, $s_{a_{j}}^{t}=\left[p_{a_{j}}^{t},v_{a_{j}}^{t}\right]$, and $s_{C}^{t}=\left[p_{C}^{t},v_{C}^{t},E_{C}^{t}\right]$ denote the states of defender *i*, attacker *j*, and the GMT, respectively.

(3) Joint Action Space (A): The joint action is $a^{t}=\left(a_{d_{1}}^{t},\ldots ,a_{d_{N}}^{t},a_{C}^{t}\right)\in A$. Here, $a_{d_{i}}^{t}\in R^{3}$ is the 3D acceleration of defender $d_{i}$, and $a_{C}^{t}\in R^{2}$ is the 2D acceleration of the GMT.

(4) Transition Function (*P*): The state transition probability $P(s^{t+1}|s^{t},a^{t})$ is determined by the defender and GMT dynamics, the rule-based attacker dynamics, environmental disturbances ω, and the stochastic generation process of new attackers.

(5) Joint Observation Space (Ω) and Observation Function (*O*): Each agent i∈I has a local observation set $\Omega^{i}$. The joint observation space is defined as the Cartesian product of all agents’ observation sets: $\Omega =\Omega_{d_{1}}\times \ldots \times \Omega_{d_{N}}\times \Omega_{C}.$ The observation function O:S×A→Δ(Ω) maps a state–action pair to a distribution over joint observations $o^{t}=\left(o_{d_{1}}^{t},\ldots ,o_{d_{N}}^{t},o_{C}^{t}\right)$. In our setting, each agent’s observation is a deterministic function of the global state:

(9) $$\begin{array}{c} o_{d_{i}}^{t}=O_{d_{i}}\left(s^{t}\right),\;o_{C}^{t}=O_{C}\left(s^{t}\right). \end{array}$$

Thus, *O* reduces to a deterministic mapping, which can be expressed as a Dirac delta distribution:

(10) $$\begin{array}{c} O\left(o^{t}|s^{t},a^{t}\right)=\delta \left(o^{t}-\left(O_{d_{1}}\left(s^{t}\right),\ldots ,O_{d_{N}}\left(s^{t}\right),O_{C}\left(s^{t}\right)\right)\right), \end{array}$$

where δ(·) denotes the Dirac delta function.

(6) Reward Function (R): Each learning agent i∈I is assigned an individual reward function $R_{i}$, forming the reward vector $R=(R_{1},R_{2},\ldots ,R_{N},R_{C})$. At each time step *t*, agent *i* receives its own reward $r_{i}^{t}=R_{i}\left(s^{t},a^{t}\right)$, which is designed to align its local objectives with the global team goals.

(7) Discount Factor (γ): The discount factor γ∈[0,1) is used to compute the cumulative return.

## 3. Hierarchical Heterogeneous MAPPO Algorithm Framework

**Figure 2:**
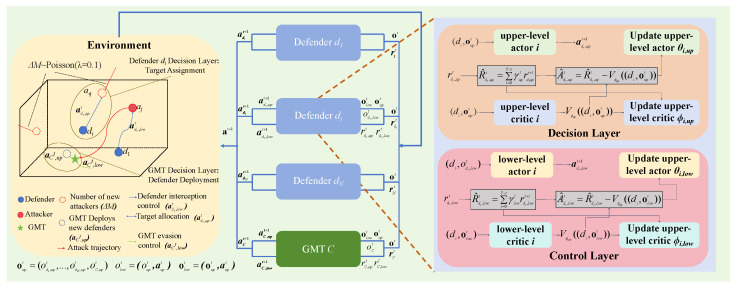
The overall framework of the proposed hierarchical heterogeneous MAPPO algorithm.

To solve the POSG formulated, which is characterized by heterogeneous agents, partial observability, and a dual-objective optimization task, we propose a Hierarchical Heterogeneous Multi-Agent Proximal Policy Optimization (HH-MAPPO) framework (Figure 2). This framework features a novel two-level policy structure integrated with role-aware embedding (RAE). The upper-level policy handles task allocation and resource management, while the lower-level policy executes fine-grained motion control. This decoupling, combined with role-aware mechanisms, directly addresses the core challenges of dynamic agent quantities and strategy homogenization.

### 3.1. Overall Architecture

The overall architecture of the proposed HH-MAPPO, illustrated in Figure 2, comprises two primary tiers. The first tier is the upper (decision) layer. It is responsible for task allocation and planning. Specifically, for each defender $d_{i}$, this layer outputs a target assignment by selecting one attacker from the set *A* to intercept. Concurrently, for the GMT *C*, it outputs a discrete decision regarding the number of additional defenders $N_{\mathrm{new}}$ to deploy. The second tier is the lower (control) layer, which handles continuous motion control. Based on the assignment from the upper layer, this layer takes the assigned target’s state and outputs precise, continuous acceleration commands $a_{d_{i}}^{t}$ or $a_{C}^{t}$ for defenders and the GMT, respectively.

### 3.2. Policy and Value Network Design

#### 3.2.1. Input Space and Observation Filtering

The design of the observation input is critical for handling the partial observability and dynamic dimensionality defined in the POSG. At time step *t*, the upper-level observation $o_{\mathrm{up}}^{t}$ is constructed as a fixed-dimensional vector, denoted as

(11) $$\begin{array}{c} o_{\mathrm{up}}^{t}=\left({\left\{o_{d_{i},\mathrm{up}}^{t}\right\}}_{i=1}^{N},o_{C,\mathrm{up}}^{t}\right),o_{d_{i},\mathrm{up}}^{t}=\left(p_{d_{i}}^{t},\Delta_{ij}^{t},p_{a_{j}}^{t},E_{d_{i}}^{t}\right), \end{array}$$

where $\Delta_{ij}^{t}=p_{d_{i}}^{t}-p_{a_{j}}^{t}$ denotes their coordinate difference at time *t*. This observation is based on the target attacker $a_{j}$ that was selected by $d_{i}$’s upper-level policy network at the previous step t−1. Note, at the initial time step (t=0), the target attacker $a_{j}$ for each defender is determined by the Hungarian algorithm based on the initial positions. When the corresponding defender does not exist (i.e., *i* exceeds the number of current defenders), the terms $o_{d_{i},\mathrm{up}}^{t}$ is padded with zeros. This design essentially acts as an observation filter: it retains only the state of the matched attacker $a_{j}$ for each $d_{i}$, thereby resolving the issue of observation dimension fluctuation caused by varying numbers of attackers. For the lower-level actor, the input is formed by concatenating the fixed-dimensional observation $o_{d_{i},\mathrm{up}}^{t}$ with the upper-level actor output $a_{d_{i},\mathrm{up}}^{t}$, i.e., $o_{d_{i},\mathrm{low}}^{t}=\left(o_{d_{i},\mathrm{up}}^{t},a_{d_{i},\mathrm{up}}^{t}\right)$. This ensures that the lower-level policy operates on a fixed-dimensional input while remaining consistent with the target assigned by the upper-level network.

The observation $o_{C,\mathrm{up}}^{t}$ of GMT (*C*) at time *t* is defined as follows:

(12) $$\begin{array}{c} o_{C,\mathrm{up}}^{t}=\left(p_{C}^{t},p_{a_{k}}^{t},d_{a_{k}}^{t},m^{t},p_{c_{a}}^{t},{\bar{d}}_{a}^{t},\sigma_{a}^{t},E_{C}^{t}\right). \end{array}$$

Here, $p_{a_{k}}^{t}$ denotes the 3D coordinates of attacker $a_{k}$ (the GMT’s closest attacker); $d_{a_{k}}^{t}=∥p_{C}^{t}-p_{a_{k}}^{t}∥$ denotes the Euclidean distance between the GMT and $a_{k}$; and $m^{t}$ denotes the total number of attackers at time *t*. The attackers’ centroid coordinates are defined as follows:

(13) $$\begin{array}{c} p_{c_{a}}^{t}=\left(\frac{1}{m^{t}}\sum_{j=1}^{m^{t}}x_{a_{j}}^{t},\frac{1}{m^{t}}\sum_{j=1}^{m^{t}}y_{a_{j}}^{t},\frac{1}{m^{t}}\sum_{j=1}^{m^{t}}z_{a_{j}}^{t}\right); \end{array}$$

${\bar{d}}_{a}^{t}$ denotes the average distance between the GMT and all attackers at time *t*:

(14) $$\begin{array}{c} {\bar{d}}_{a}^{t}=\frac{1}{m^{t}}\sum_{j=1}^{m^{t}}∥p_{C}^{t}-p_{a_{j}}^{t}∥ \end{array}$$

and $\sigma_{a}^{t}$ quantifies the spatial dispersion of attackers at time *t*:

(15) $$\begin{array}{c} \sigma_{a}^{t}=\frac{1}{m^{t}}\sum_{j=1}^{m^{t}}∥d_{a_{j}}^{t}-{\bar{d}}_{a}^{t}∥. \end{array}$$

Subsequently, the lower-level actor of the GMT receives an expanded input concatenation $o_{C,\mathrm{low}}^{t}=\left(o_{C,\mathrm{up}}^{t},a_{C,\mathrm{up}}^{t}\right)$. Here, $a_{C,\mathrm{up}}^{t}$ represents the upper-level action (specifically, the command determining how many new defenders to deploy), ensuring the lower-level policy’s behavior is conditioned on the strategic decision made at the upper-level.

#### 3.2.2. Role-Aware Embedding

**Figure 3:**
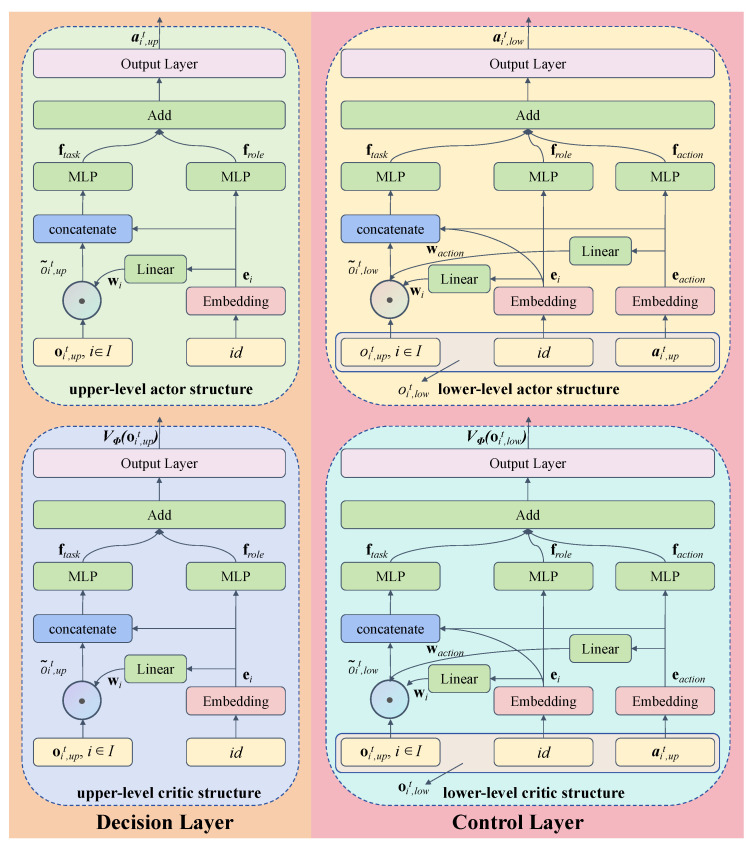
The upper-layer and lower-layer network structures.

To mitigate policy homogenization in shared networks and foster role differentiation among homogeneous defenders, we introduce a RAE mechanism. The upper-layer and lower-layer network structures are shown in Figure 3. Each agent i∈I is assigned a unique identifier id, which is mapped to a role embedding vector $e_{i}=\mathrm{Embedding}\left(id\right)\in R^{d}$.

For the upper-level network, the processing of $e_{i}$ is as follows: it is transformed by a linear layer into a modulation vector $w_{i}$ for observation conditioning via ${\tilde{o}}_{i,up}=o_{i,up}⊙w_{i}$. The modulated observation $\left[{\tilde{o}}_{i,up};e_{i}\right]$ is then fed into a shared MLP to extract task-oriented features $f_{\mathrm{task}}$. In parallel, $e_{i}$ is processed by a separate lightweight MLP to obtain role-specific features $f_{\mathrm{role}}$. The final upper-level action is given by

(16) $$\begin{array}{c} a_{i,\mathrm{up}}=f_{\mathrm{out}}\left(f_{\mathrm{task}}+f_{\mathrm{role}}\right). \end{array}$$

The lower-level network adopts a similar architecture but with an additional branch for processing the upper-level action. Specifically, the discrete action $a_{i,\mathrm{up}}$ is mapped to an action embedding $e_{\mathrm{action}}=\mathrm{Embedding}\left(a_{i,\mathrm{up}}\right)$ through a dedicated embedding layer. This action embedding $e_{\mathrm{action}}$ is then processed in a manner analogous to the role embedding $e_{i}$. Specifically, it generates a modulation vector $w_{\mathrm{action}}$ and contributes to the task features $f_{\mathrm{task}}$ and action-specific features $f_{\mathrm{action}}$ through the same pipeline structure.

The final lower-level policy output synthesizes all three feature streams:

(17) $$\begin{array}{c} a_{i,\mathrm{low}}=f_{\mathrm{out}}\left(f_{\mathrm{task}}+f_{\mathrm{role}}+f_{\mathrm{action}}\right). \end{array}$$

This design ensures that the lower-level control policy is conditioned on both the agent’s persistent role identity and the specific strategic intention from the upper level, enabling effective hierarchical decision-making.

#### 3.2.3. Action Space and Network Output

Upper-Level Outputs: A defender’s upper-level policy network outputs a discrete action $a_{d_{i},\mathrm{up}}^{t}$, corresponding to the index of its assigned target attacker selected from the set of currently alive attackers. That is, $a_{d_{i},\mathrm{up}}^{t}=q$ denotes that defender $d_{i}$ is assigned to intercept attacker $a_{q}$. Similarly, the GMT’s upper-level policy outputs a discrete action $a_{C,\mathrm{up}}^{t}$ chosen from a finite set of possible defender increments, which directly specifies the number of additional defenders to deploy at time *t*.

Lower-Level Outputs: The defender’s lower-level actor outputs a three-dimensional vector representing the mean of a Gaussian distribution for its acceleration $a_{d_{i},\mathrm{low}}^{t}\in R^{3}$. The GMT’s lower-level actor outputs a two-dimensional vector for its acceleration $a_{C,\mathrm{low}}^{t}\in R^{2}$. The standard deviation is a separate, learnable parameter.

#### 3.2.4. Centralized Critic Network

The centralized critic $V_{\phi }\left(o^{t}\right)$ estimates the state-value function. During training, it uses the joint observations $o^{t}$ to compute a baseline for reducing the variance of policy gradients. Its output is a scalar representing the expected return from state $o^{t}$ under the current joint policy.

### 3.3. Training with Hierarchical Rewards

#### 3.3.1. Hierarchical Reward Design

**The global objective is to achieve a Pareto optimal balance between interception rate and energy consumption, meaning to maximize the interception rate under a given energy constraint.** We design a two-level reward mechanism, where the upper-level reward $R_{\mathrm{up}}$ guides strategic coordination (target assignment and resource deployment), and the lower-level reward guides motion control.

##### Upper-Level Reward

$R_{\mathrm{up}}$ integrates three components to balance effective coverage, resource economy, and task coordination:

(18) $$R_{\mathrm{up}}=\alpha R_{\mathrm{match}}+\beta R_{\mathrm{num}}-\kappa R_{\mathrm{penalty}},$$

where α,β,κ>0 are weighting coefficients.

**Matching rationality (**$R_{\mathrm{match}}$**).** This component rewards high-quality, non-redundant defender–attacker pairings. It is the product of an average pairing-quality score and a non-redundancy factor:

(19) $$R_{\mathrm{match}}=\underset{\mathrm{Average}\;\mathrm{pairing}\;\mathrm{quality}}{\underset{︸}{\left(\frac{1}{\left|P\right|}\sum_{(d_{i},a_{j})\in P}S\left(d_{i},a_{j}\right)\right)}}\times \underset{\mathrm{Non}-\mathrm{redundancy}\;\mathrm{factor}}{\underset{︸}{\left(1-\frac{T_{\mathrm{repeated}}}{M_{\mathrm{alive}}}\right)}}.$$

P is the set of valid pairs between alive defenders and alive attackers, $T_{\mathrm{repeated}}$ counts repeated matches to the same alive attacker, and $M_{\mathrm{alive}}$ is the total number of alive attackers.

The pairing quality score $S(d_{i},a_{j})$ evaluates the suitability of defender $d_{i}$ to intercept attacker $a_{j}$. It combines three normalized ($S\left(d_{i},a_{j}\right)\in \left[0,1\right]$) metrics:

(20) $$S\left(d_{i},a_{j}\right)=\omega_{c}S_{\mathrm{cover}}+\omega_{t}S_{\mathrm{threat}}+\omega_{d}S_{\mathrm{dist}},$$

where $\omega_{c},\omega_{t},\omega_{d}$ are weights.

Coverage capability $S_{\mathrm{cover}}$ reflects whether $d_{i}$ possesses sufficient energy to reach and intercept $a_{j}$. Let the energy required be

(21) $$E_{\mathrm{req}}\left(d_{i},a_{j}\right)=\frac{∥p_{d_{i}}-p_{a_{j}}{∥}^{2}}{2\chi_{d}}+E_{d}^{\min}.$$

Then

(22) $$S_{\mathrm{cover}}=\max\;\left(0,\;1-\frac{E_{\mathrm{req}}\left(d_{i},a_{j}\right)}{E_{d_{i}}}\right).$$

Threat level $S_{\mathrm{threat}}$ is inversely proportional to the distance between attacker $a_{j}$ and the GMT:

(23) $$S_{\mathrm{threat}}=\max\;\left(0,\;1-\frac{d_{\mathrm{GMT}}\left(a_{j}\right)}{D_{\mathrm{threat}}}\right),$$

where $d_{\mathrm{GMT}}\left(a_{j}\right)$ is the Euclidean distance and $D_{\mathrm{threat}}$ a threshold beyond which the threat is considered negligible.

Relative distance $S_{\mathrm{dist}}$ favors closer defender–attacker pairs:

(24) $$S_{\mathrm{dist}}=\max\;\left(0,\;1-\frac{∥p_{d_{i}}-p_{a_{j}}∥}{D_{\mathrm{match}}}\right),$$

with $D_{\mathrm{match}}$ the maximum distance for a valid match.

**Quantity appropriateness (**$R_{\mathrm{num}}$**).** This component guides the GMT’s decision on how many new defenders $N_{\mathrm{new}}$ to deploy:

(25) $$R_{\mathrm{num}}=S_{\mathrm{need}}\cdot S_{\mathrm{moderate}}.$$

Necessity score $S_{\mathrm{need}}$ is designed to strongly incentivize deployment when coverage is incomplete:

(26) $$S_{\mathrm{need}}=\left\{\begin{array}{cc} 1.0, & C_{\mathrm{full}}<1,\\ 0.2, & C_{\mathrm{full}}=1. \end{array}\right.$$

An alive attacker is considered covered if at least one alive defender has sufficient energy to intercept it (i.e., $E_{d_{i}}\geq E_{\mathrm{req}}\left(d_{i},a\right)$ for that attacker *a*). Let $M_{\mathrm{covered}}$ be the number of such covered attackers. The coverage completeness is then defined as $C_{\mathrm{full}}=M_{\mathrm{covered}}/M_{\mathrm{alive}}$.

Moderation score $S_{\mathrm{moderate}}$ assesses how closely $N_{\mathrm{new}}$ matches the minimum required number of new defenders $N_{\mathrm{req}-\min}$ (i.e., the number of currently uncovered attackers). It is defined as follows:

(27) $$S_{\mathrm{moderate}}=\left\{\begin{array}{c} 1.0,\;N_{\mathrm{req}-\min}=0\;\mathrm{and}\;N_{\mathrm{new}}=0,\\ 0.5,\;N_{\mathrm{req}-\min}=0\;\mathrm{and}\;N_{\mathrm{new}}>0,\\ \max\;\left(0,\;1-\frac{|N_{\mathrm{new}}-N_{\mathrm{req}-\min}|}{N_{\mathrm{req}-\min}+1}\right),\;N_{\mathrm{req}-\min}>0. \end{array}\right.$$

Under full coverage ($N_{\mathrm{req}-\min}=0$), any unnecessary deployment is penalized. When additional defenders are needed ($N_{\mathrm{req}-\min}>0$), the score linearly decreases with the absolute deviation, normalized by $N_{\mathrm{req}-\min}+1$ to maintain a positive reward for under-deployment.

**Penalty term (**$R_{\mathrm{penalty}}$**).** This term directly penalizes three undesirable strategic outcomes:

(28) $$\begin{array}{c} R_{\mathrm{penalty}}=\zeta_{c}I\left(C_{\mathrm{full}}<1\right)\;+\;\zeta_{e}I\left(N_{\mathrm{new}}>N_{\mathrm{req}-\min}\right)\;+\;\zeta_{r}I\left(T_{\mathrm{repeated}}>0\right), \end{array}$$

where I(·) is the indicator function, and $\zeta_{c},\zeta_{e},\zeta_{r}$ are penalty coefficients. The penalties respectively discourage incomplete coverage, excessive deployment, and redundant matches.

##### Individual Credit Assignment

To address the multi-agent credit assignment problem, the global reward $R_{\mathrm{up}}$ is distributed to each agent i∈I via a contribution weight. To stabilize training and prevent extreme gradient updates, the resulting reward is clipped to a fixed range $\left[R_{\min},R_{\max}\right]$, yielding the individual upper-level reward:

(29) $$r_{i,\mathrm{up}}^{t}=\mathrm{clip}\;\left(R_{\mathrm{up}}\cdot w_{i},\;R_{\min},\;R_{\max}\right).$$

The clipping bounds $R_{\min}$ and $R_{\max}$ are hyperparameters set empirically.

In the following, we specify the contribution weight for each agent type: for a defender $d_{i}$ we denote it as $w_{d_{i}}$, and for the GMT we denote it as $w_{C}$. Both are special cases of the general $w_{i}$ in Equation (29).

**Defender weight** $w_{d_{i}}$**.** For defender $d_{i}$ intercepting attacker $a_{j}$, its contribution weight combines three factors:

(30) $$w_{d_{i}}=\tau_{c}w_{\mathrm{cover}}+\tau_{t}w_{\mathrm{threat}}+\tau_{e}w_{\mathrm{energy}},$$

with $\tau_{c},\tau_{t},\tau_{e}>0$ balancing the factors.

Coverage uniqueness $w_{\mathrm{cover}}$ rewards a defender $d_{i}$ that is exclusively capable of intercepting its target attacker $a_{j}$:

(31) $$w_{\mathrm{cover}}=\left\{\begin{array}{cc} 1.0, & d_{i}\mathrm{is}\;\mathrm{the}\;\mathrm{sole}\;\mathrm{defender}\;\mathrm{with}\;E_{d_{i}}\geq E_{\mathrm{req}}\left(d_{i},a_{j}\right)\;\mathrm{for}\;\mathrm{attacker}\;a_{j},\\ 0.3, & d_{i}\;\mathrm{is}\;\mathrm{one}\;\mathrm{of}\;\mathrm{several}\;\mathrm{such}\;\mathrm{defenders},\\ 0.0, & \mathrm{otherwise}. \end{array}\right.$$

Threat proximity $w_{\mathrm{threat}}$ is defined identically to $S_{\mathrm{threat}}$ in Equation (23):

(32) $$w_{\mathrm{threat}}=\max\;\left(0,\;1-\frac{d_{\mathrm{GMT}}\left(a_{j}\right)}{D_{\mathrm{threat}}}\right),$$

where $d_{\mathrm{GMT}}\left(a_{j}\right)$ is the distance between attacker $a_{j}$ and the GMT.

Energy efficiency $w_{\mathrm{energy}}$ gives a bonus when defender $d_{i}$ has ample energy margin:

(33) $$w_{\mathrm{energy}}=\left\{\begin{array}{cc} 1.0, & E_{d_{i}}/E_{\mathrm{req}}\left(d_{i},a_{j}\right)\geq 1.2,\\ 0.8, & 1.0\leq E_{d_{i}}/E_{\mathrm{req}}\left(d_{i},a_{j}\right)<1.2,\\ 0.0, & \mathrm{otherwise}. \end{array}\right.$$

**GMT weight** $w_{C}$**.** The GMT’s contribution weight reflects the quality of its deployment decision:

(34) $$w_{C}=\eta_{n}w_{\mathrm{need}}+\eta_{m}w_{\mathrm{moderate}},$$

where $\eta_{n},\eta_{m}>0$ are weights. The factors $w_{\mathrm{need}}$ and $w_{\mathrm{moderate}}$ are defined analogously to $S_{\mathrm{need}}$ and $S_{\mathrm{moderate}}$ in Equations (26) and (27):

(35) $$w_{\mathrm{need}}=\left\{\begin{array}{cc} 1.0, & C_{\mathrm{full}}<1,\\ 0.2, & C_{\mathrm{full}}=1, \end{array}\right.$$

(36) $$w_{\mathrm{moderate}}=\left\{\begin{array}{c} 1.0,\;N_{\mathrm{req}-\min}=0\;\mathrm{and}\;N_{\mathrm{new}}=0,\\ 0.5,\;N_{\mathrm{req}-\min}=0\;\mathrm{and}\;N_{\mathrm{new}}>0,\\ \max\;\left(0,\;1-\frac{|N_{\mathrm{new}}-N_{\mathrm{req}-\min}|}{N_{\mathrm{req}-\min}+1}\right),\;N_{\mathrm{req}-\min}>0. \end{array}\right.$$

##### Lower-Level Reward

The lower-level reward governs motion control: it directs defenders to close in on their assigned attackers and guides the GMT to evade threats. For each agent i∈I, the motion reward $r_{i,\mathrm{low}}^{t}$ is defined separately.

For the defender (i∈D), the motion reward $r_{i,\mathrm{low}}^{t}$ is as follows:

(37) $$r_{i,\mathrm{low}}^{t}=∥p_{i}^{t-1}-p_{a_{q}}^{t-1}∥-∥p_{i}^{t}-p_{a_{q}}^{t-1}∥,$$

where $a_{q}$ is the target attacker index for the defender $d_{i}$ output by the upper-level policy. The reward is positive when the defender moves closer to its target attacker.

For GMT (i∈C), the motion reward $r_{i,\mathrm{low}}^{t}$ is as follows:

(38) $$r_{i,\mathrm{low}}^{t}=∥p_{i}^{t}-p_{a_{k}}^{t-1}∥-∥p_{i}^{t-1}-p_{a_{k}}^{t-1}∥,$$

where $a_{k}$ is the most threatening attacker (e.g., the closest one). The reward is positive when the GMT moves away from that threat.

##### Hyperparameter Summary

All weighting coefficients, penalty coefficients, distance thresholds, and clipping bounds are summarized in Table 2. Their default values are determined empirically and kept fixed throughout the experiments.

#### 3.3.2. Hierarchical MAPPO Training Objectives

The hierarchical reward design is integrated into a MAPPO framework with separate networks for upper-level and lower-level policies. Both policy levels are optimized jointly in an end-to-end manner.

**Upper-level optimization**: For the upper-level policy, each agent (defender or GMT) receives an individual reward $r_{i,\mathrm{up}}^{t}$ derived from the global upper-level reward $R_{\mathrm{up}}$ through the contribution weight mechanism described in Section 3.3.1. The discounted return for agent *i* at time *t* is computed as follows:

(39) $$\begin{array}{c} {\hat{R}}_{i,\mathrm{up}}^{t}=\sum_{l=0}^{T-t}\gamma_{\mathrm{up}}^{l}r_{i,\mathrm{up}}^{t+l}, \end{array}$$

where $\gamma_{\mathrm{up}}$ is the discount factor for the upper level. The upper-level actor parameters $\theta_{\mathrm{up}}$ are updated by maximizing the PPO-Clip objective:

(40) $$\begin{array}{cc} L_{\mathrm{up}}^{\mathrm{CLIP}}\left(\theta_{\mathrm{up}}\right) & =E_{t}\left[\min\left(\rho_{t}\left(\theta_{\mathrm{up}}\right){\hat{A}}_{i,\mathrm{up}}^{t},\mathrm{clip}\left(\rho_{t}\left(\theta_{\mathrm{up}}\right),1-\epsilon ,1+\epsilon \right){\hat{A}}_{i,\mathrm{up}}^{t}\right)\right], \end{array}$$

where $\rho_{t}\left(\theta_{\mathrm{up}}\right)=\pi_{\theta_{\mathrm{up}}}\left(a_{i,\mathrm{up}}^{t}|o_{i,\mathrm{up}}^{t}\right)/\pi_{\theta_{\mathrm{upper},\mathrm{old}}}\left(a_{i,\mathrm{up}}^{t}|o_{i,\mathrm{up}}^{t}\right)$, ϵ is a clipping hyperparameter, and ${\hat{A}}_{i,\mathrm{up}}^{t}={\hat{R}}_{i,\mathrm{up}}^{t}-V_{\phi_{\mathrm{up}}}\left(o_{\mathrm{up}}^{t}\right)$ is the advantage estimate computed using the upper-level centralized critic $V_{\phi_{\mathrm{up}}}$. The upper-level critic parameters $\phi_{\mathrm{up}}$ are updated by minimizing the value function loss:

(41) $$\begin{array}{c} L_{\mathrm{up}}^{\mathrm{VF}}\left(\phi_{\mathrm{up}}\right)=E_{t}\left[{\left(V_{\phi_{\mathrm{up}}}\left(o_{\mathrm{up}}^{t}\right)-{\hat{R}}_{i,\mathrm{up}}^{t}\right)}^{2}\right]. \end{array}$$

**Lower-level optimization**: For the lower-level policy, each agent receives the motion reward $r_{i,\mathrm{low}}^{t}$ defined in Equations (37) and (38). The discounted return is as follows:

(42) $$\begin{array}{c} {\hat{R}}_{i,\mathrm{low}}^{t}=\sum_{l=0}^{T-t}\gamma_{\mathrm{low}}^{l}r_{i,\mathrm{low}}^{t+l}, \end{array}$$

with $\gamma_{\mathrm{low}}$ being the lower-level discount factor. The lower-level actor parameters $\theta_{\mathrm{low}}$ are updated similarly using the PPO-Clip objective:

(43) $$\begin{array}{cc} L_{\mathrm{low}}^{\mathrm{CLIP}}\left(\theta_{\mathrm{low}}\right) & =E_{t}\left[\min\left(\rho_{t}\left(\theta_{\mathrm{low}}\right){\hat{A}}_{i,\mathrm{low}}^{t},\mathrm{clip}\left(\rho_{t}\left(\theta_{\mathrm{low}}\right),1-\epsilon ,1+\epsilon \right){\hat{A}}_{i,\mathrm{low}}^{t}\right)\right], \end{array}$$

where $\rho_{t}\left(\theta_{\mathrm{low}}\right)=\pi_{\theta_{\mathrm{low}}}\left(a_{i,\mathrm{low}}^{t}|o_{i,\mathrm{low}}^{t}\right)/\pi_{\theta_{\mathrm{low},\mathrm{old}}}\left(a_{i,\mathrm{low}}^{t}|o_{i,\mathrm{low}}^{t}\right)$, and ${\hat{A}}_{i,\mathrm{low}}^{t}={\hat{R}}_{i,\mathrm{low}}^{t}-V_{\phi_{\mathrm{low}}}\left(o_{\mathrm{low}}^{t}\right)$ uses the lower-level centralized critic $V_{\phi_{\mathrm{low}}}$. The corresponding critic loss is as follows:

(44) $$\begin{array}{c} L_{\mathrm{low}}^{\mathrm{VF}}\left(\phi_{\mathrm{low}}\right)=E_{t}\left[{\left(V_{\phi_{\mathrm{low}}}\left(o_{\mathrm{low}}^{t}\right)-{\hat{R}}_{i,\mathrm{low}}^{t}\right)}^{2}\right]. \end{array}$$

This hierarchical training approach enables agents to learn coordinated behaviors at both strategic (target assignment and resource management) and tactical (movement control) levels, with each level’s policy optimized for its specific objectives while contributing to the overall mission success. The complete training procedure of the HH-MAPPO framework is summarized in Algorithm 1.
**Algorithm 1:** HH-MAPPO for dynamic TAD defense
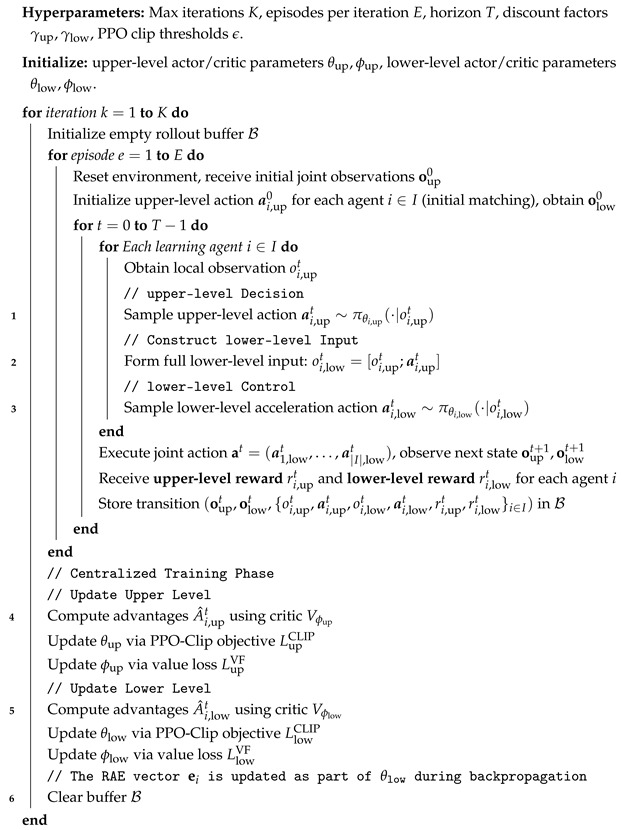


## 4. Experimental Results and Analysis

### 4.1. Environment and Parameters

A closed, bounded 3D simulation environment (20 × 20 × 20) is developed in this study. Attackers are generated dynamically throughout an episode following a Poisson process with an arrival rate λ=0.1 per time step, entering the environment from random positions on the boundary. Each defender and the GMT possess limited initial energy, and their motion energy consumption follows the quadratic model defined in Section 2.1. The game terminates when all attackers are intercepted or any attacker captures the GMT.

The neural networks use two fully connected layers of 256 units with Tanh activation. We train for a total of $1\times 10^{7}$ steps, with an episode horizon of 200 steps. The Adam optimizer is adopted for both the actor and critic networks. The learning rates are set to $1\times 10^{-5}$ for the actor network and $1\times 10^{-4}$ for the critic network.

For every method, we conduct five independent training runs with different random seeds. After convergence, each run is evaluated over 100 test episodes, and the average performance is recorded. We report the mean and standard deviation across the five runs.

### 4.2. Baseline and Ablation Methods

To comprehensively evaluate the proposed Hierarchical Heterogeneous MAPPO (HH-MAPPO), we compare it against several strong baselines and conduct critical ablation studies. All experiments were implemented in Python 3.11.5.

- **Heterogeneous MAPPO (HMAPPO)**: This scheme employs a single-layer control architecture, where the HMAPPO algorithm is directly used to generate motion policies for both the defenders and the GMT. At its core, it implicitly learns multi-agent coordination and target selection strategies during training through a carefully designed reward signal. This signal calculates the potential interaction reward between each defender and all attackers and selects the maximum value to guide learning.
- **Hungarian+HMAPPO**: This scheme adopts a two-layer decision-making architecture to achieve explicit task coordination. The upper layer utilizes the Hungarian algorithm to compute the optimal one-to-one interception matching between defenders and attackers, generating deterministic task assignment instructions. Based on these fixed instructions, each defender in the lower layer then employs an independent HMAPPO network to control its own motion, focusing solely on intercepting its specifically assigned target. Consequently, the coordination logic is explicitly dictated by the upper-layer algorithm, rather than being learned from rewards.
- **Auction+HMAPPO**: Similar to Hungarian+HMAPPO but employs an auction algorithm for the upper-layer assignment, testing a different rule-based coordination strategy.
- **HH-MAPPO (w/o RAE)**: An ablation variant that removes the Role-Aware Embedding (RAE) module. This directly tests RAE’s contribution to policy diversification.
- **HH-MAPPO (w/o RAE, w/ Independent Output Layer)**: An alternative method for policy differentiation, where each defender has a unique final output layer within a shared backbone network. This contrasts with our RAE approach.
- **HH-MAPPO (w/ RAE and Independent Output Layer)**: A combined-method that integrates both the RAE module and independent final output layers for each defender. This design is used to investigate the potential complementary effects and assess whether the combination of RAE and independent layers yields superior policy diversification and performance compared to using either mechanism alone.

### 4.3. Evaluation Metrics

We employ the following metrics for quantitative evaluation:

**Interception Rate (IR)**: IR=IA/TA, where IA and TA are the numbers of intercepted and total attackers, respectively. This is the primary metric for defense effectiveness.

**Total Defender Energy Consumption (TE)**: $TE=\sum_{d_{i}\in D}\left(E_{d_{i},\mathrm{init}}-E_{d_{i},\mathrm{final}}\right)$. This measures the energy efficiency of the defense team.

**Sequence-Based Action Dissimilarity (SBAD)**: To quantitatively measure policy homogeneity, we define $SBAD=1-\bar{\mathrm{Sim}}$, where $\bar{\mathrm{Sim}}$ is the average pairwise cosine similarity of normalized action sequences among defenders over an episode. A higher SBAD indicates greater policy differentiation.

(45) $$\bar{\mathrm{Sim}}=\frac{2}{N(N-1)}\sum_{i=1}^{N-1}\sum_{j=i+1}^{N}\frac{a_{d_{i}}\cdot a_{d_{j}}}{∥a_{d_{i}}∥∥a_{d_{j}}∥}$$

Here, $a_{d_{i}}=\left[a_{d_{i}}^{\left(1\right)},\ldots ,a_{d_{i}}^{\left(T\right)}\right]$ is the acceleration sequence vector for defender $d_{i}$.

### 4.4. Comprehensive Experimental Analysis

To comprehensively validate the effectiveness of the HH-MAPPO framework, we conducted a systematic experimental analysis encompassing benchmark performance testing, core component ablation studies, and robustness evaluation.

**Figure 4:**
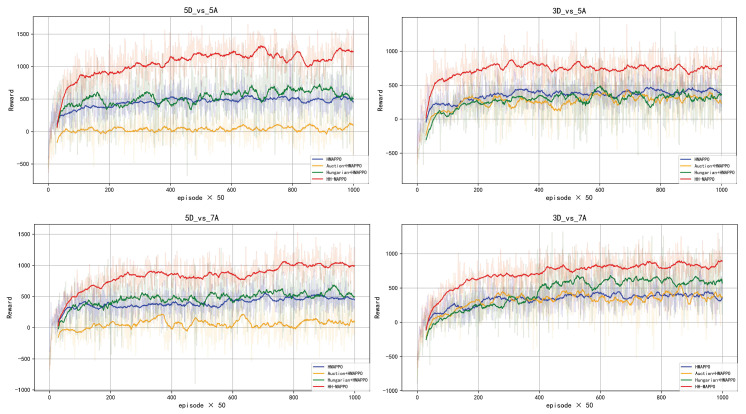
Reward curves of different methods across various scenarios.

**Figure 5:**
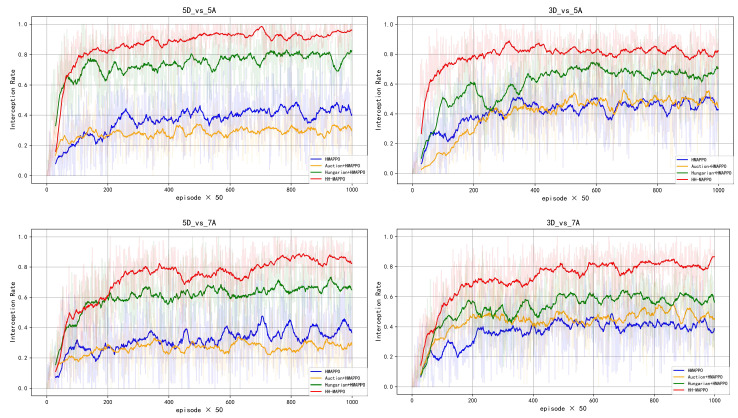
Interception rate curves of different methods across various scenarios.

The overall performance comparison (Table 3) indicates that HH-MAPPO achieved the highest Interception Rate (IR) with a significant advantage across all four adversarial scenarios: 3D_vs_5A (max three defenders vs. five attackers), 3D_vs_7A, 5D_vs_5A, and 5D_vs_7A (max five defenders vs. seven attackers). A key finding is that its learnable upper-level policy delivered a substantial performance leap compared to the fixed-rule method (Hungarian) (IR: 93.64% vs. 77.76%). This confirms the superiority of learnable hierarchical decision-making: fixed rules fail to achieve dynamic coordination between GMT and defenders, easily leading to resource misallocation. In contrast, our policy can optimize target assignment and defender unit generation in real-time based on the global situation, demonstrating exceptional strategic flexibility. From the training dynamics perspective (Figure 4), the end-to-end hierarchical training of HH-MAPPO achieved faster convergence, avoiding the suboptimality and latency caused by the decoupling of decision and control in rule-based hierarchical methods. Meanwhile, the interception rate curves (Figure 5) show that our method maintains a clear advantage over all baselines across the four scenarios.

**Figure 6:**
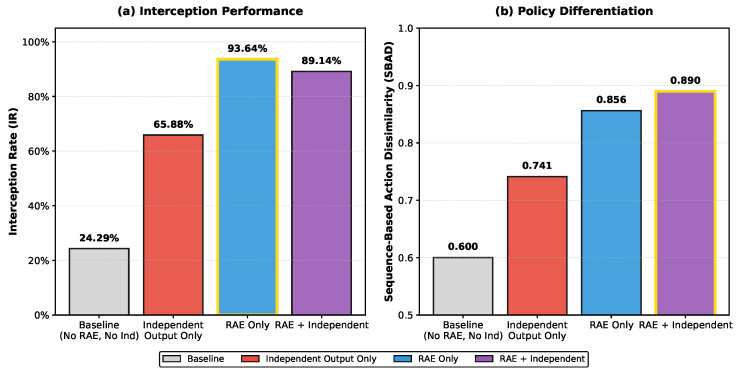
Performance comparison between RAE and independent output layer methods. (**a**) Interception performance. (**b**) Policy differentiation.

The validation of RAE’s effectiveness was accomplished through ablation experiments in the 5D_vs_5A scenario. As shown in Figure 6, removing RAE led to a significant drop in IR (Baseline w/o RAE) accompanied by strategy homogenization. Specifically, the SBAD metric was low, indicating convergent defender behaviors that triggered coverage blind spots and mobility redundancy. Conversely, HH-MAPPO achieved a high SBAD value, demonstrating that RAE effectively fosters specialized role differentiation.

**Figure 7:**
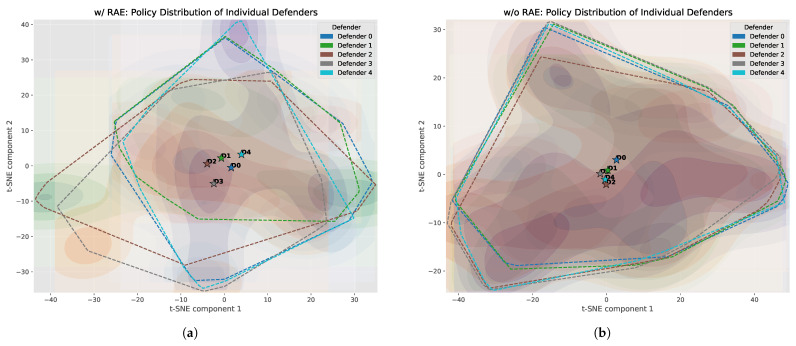
t-SNE visualization of defender action distributions. (**a**) With RAE. (**b**) Without RAE.

**Figure 8:**
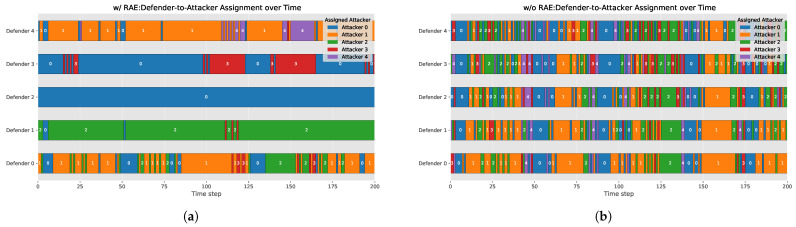
Gantt charts of defender-to-attacker assignments over time. (**a**) With RAE. (**b**) Without RAE.

To further visually demonstrate this differentiation, we performed t-SNE dimensionality reduction on the defenders’ action sequences (Figure 7) and plotted Gantt charts of target assignments (Figure 8). In the t-SNE plots, the w/ RAE configuration shows five well-separated convex hulls (each representing the action distribution of one defender) and widely scattered centroids, indicating distinct motion patterns across defenders. In contrast, the w/o RAE configuration exhibits largely overlapping convex hulls and clustered centroids, implying homogeneous behaviors. The Gantt charts further reveal that w/ RAE leads to diverse and temporally stable assignments. Specifically, at any given time step, different defenders typically intercept different attackers, and switches between targets are infrequent. However, w/o RAE often causes multiple defenders to chase the same attacker simultaneously, reflecting a lack of coordinated role differentiation.

To further demonstrate RAE’s superiority over alternative diversification mechanisms, we compared it with the Independent Output Layers method (w/o RAE, w/ Independent Output Layer). RAE achieved both a higher IR (93.64% vs. 65.88%) and greater policy dissimilarity (SBAD: 0.856 vs. 0.741). This indicates that RAE is a more effective mechanism for strategy differentiation. Each agent acquires a dedicated, learnable embedding vector through the RAE mechanism, which endows it with a persistent and identifiable role identity. This persistent representation enables agents to develop complementary specialized skills. In contrast, while independent output layers offer parameter independence, they lack explicit role representation, which can easily lead to role drift. Notably, the combination of RAE and independent output layers (w/ RAE and Independent Output Layer) did not produce the anticipated synergistic effect; instead, it slightly reduced the interception rate. Specifically, although the “RAE+Independent” combination achieved the highest SBAD value (0.890), its IR (89.14%) was lower than that of the pure RAE method (93.64%). This result reveals a crucial distinction. It is the distinction between structured differentiation and unstructured divergence. In pure RAE, all defenders share the same feature extraction backbone. They develop a common understanding of the task. The distinct role embeddings provide a persistent bias. This bias operates within the shared representational space. As a result, agents develop complementary yet predictable behaviors. In the RAE plus independent output layer variant, each defender has its own output layer. These independent layers allow policies to diverge into separate function spaces. There is no shared grounding. The resulting actions show high dissimilarity, measured by a high SBAD. However, these actions become unpredictable to teammates. This unpredictability disrupts coordinated teamwork. Therefore, what matters for cooperation is not the magnitude of action dissimilarity. It is whether the differentiation is structured. Structured differentiation means the differentiation is grounded in a shared representation. This shared representation preserves inter-agent predictability.

**Figure 9:**
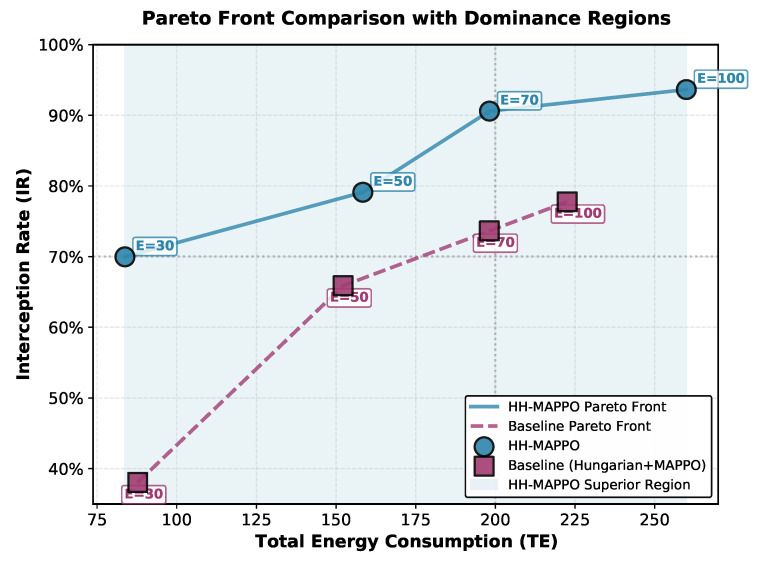
Comparative analysis of Pareto frontiers. This figure presents the performance trade-offs between the HH-MAPPO framework and the baseline method under different initial energy constraints (E = 30, 50, 70, 100). The Pareto frontier of HH-MAPPO (solid blue line) consistently outperforms that of the baseline method (dashed red line) across all tested energy levels, indicating that HH-MAPPO achieves a more favorable performance–energy trade-off at each evaluated operating point.

Adaptive capability under energy constraints was evaluated through Pareto frontier analysis in the 5D_vs_5A scenario (Figure 9). As shown, the Pareto frontier of HH-MAPPO clearly reveals the marginal trade-off relationship between the Interception Rate (IR) and Total Energy consumption (TE). More importantly, HH-MAPPO consistently achieves a higher interception rate than the baseline methods at each tested energy level (E = 30, 50, 70, 100), indicating that our approach maintains superior interception performance across different energy budgets.

This systematic advantage stems from the end-to-end hierarchical reinforcement learning framework adopted by HH-MAPPO, which offers intelligent advantages in dynamic resource scheduling and task allocation compared to the fixed-rule upper-level structure of the baseline methods. Specifically, baseline methods rely on fixed rules for target assignment and defender quantity generation, lacking consideration for the global situation and long-term effectiveness. In contrast, the upper-level policy network of HH-MAPPO learns to jointly optimize the two decisions of defender generation quantity and dynamic target assignment. This enables the algorithm to proactively adapt to energy constraints. When energy is abundant, it adopts a more aggressive defense posture. When energy is limited, it intelligently trades off interception success rate against energy consumption and makes more economical scheduling decisions. This helps avoid resource wastage on low-threat or difficult-to-intercept targets.

Quantitative analysis robustly supports the effectiveness of this mechanism: at an equivalent energy consumption level (*TE* ≈ 200), HH-MAPPO achieved a 90.59% IR, representing a 23.02% relative improvement over the baseline’s 73.64%. Under an equivalent performance requirement (*IR* = 0.7), HH-MAPPO required only 84.0 units of energy, whereas the baseline method needed 176.6 units, resulting in a significant 52.4% reduction in energy consumption. This validates the superior energy utilization efficiency of HH-MAPPO in accomplishing the same mission objectives.

Its robustness under extreme constraints is particularly outstanding. When the initial energy cap was set to 30 (the leftmost point in Figure 9), HH-MAPPO still maintained a 69.97% IR, with a unit-energy interception rate (*IR*/*TE*) as high as 8.36‰. In contrast, the baseline method, at a similar energy consumption level (*TE* = 87.76), saw its IR plummet to 38.06%. This substantial gap (an absolute IR advantage of 31.9% and a relative advantage of 83.8%) fully demonstrates the effectiveness of HH-MAPPO. Through dynamic planning via its upper-level policy network, HH-MAPPO intelligently concentrates limited resources on defending the most critical areas and targets. Consequently, it maintains considerable interception efficacy even in scenarios with severely constrained resources.

**Figure 10:**
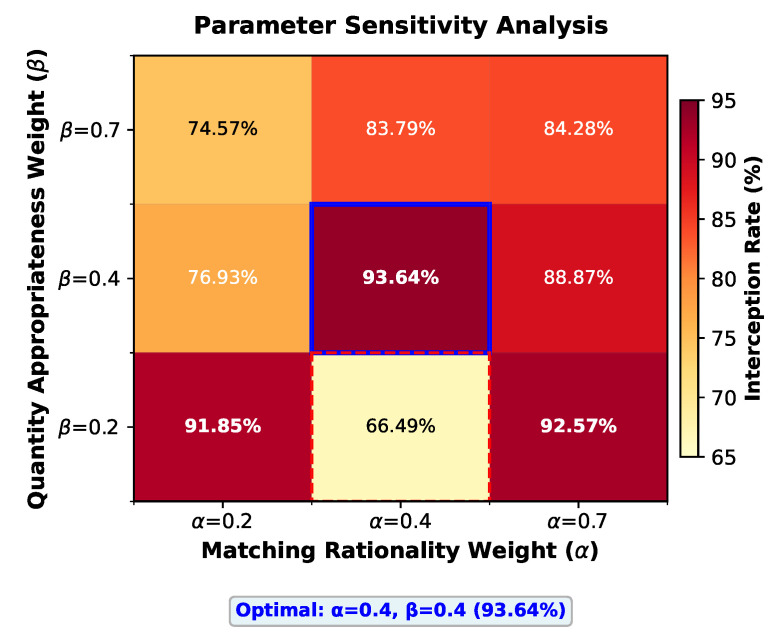
Parameter sensitivity analysis heatmap. Shows the interception rate under different combinations of α (matching rationality weight) and β (quantity appropriateness weight). The blue box marks the optimal parameter combination (α=0.4,β=0.4), achieving the highest interception rate of 93.64%. The red dashed box marks the worst-performing combination (α=0.4,β=0.2), with only 66.49%.

The matching rationality weight α and the quantity suitability weight β have a significant impact on the research objectives. Therefore, this paper focuses on analyzing the sensitivity of performance to α and β in the 5D_vs_5A scenario, with experimental results shown in Figure 10. The heatmap analysis reveals the algorithm’s sensitivity characteristics to the hyperparameters α and β. Specifically, the algorithm achieves peak performance (93.64% interception rate) when α=0.4 and β=0.4. This optimal point represents a 9.97% improvement over the average performance (83.67%) across the parameter space, validating the value of fine-grained parameter tuning. When α is fixed at 0.4, varying β from 0.2 to 0.7 leads to marked fluctuations in the interception rate (66.49% → 93.64% → 83.79%). The influence of β on performance accounts for 27.15%, significantly higher than α’s contribution of 16.71%. This indicates that a well-designed quantity regulation mechanism is crucial for cooperative defense. We further examined the sensitivity to the penalty weight κ with the optimal α=0.4 and β=0.4 fixed. When κ was increased from 0.2 to 0.4, the interception rate showed only a slight decrease from 93.64% to 91.29%, indicating robustness to moderate variations. However, a larger increase to κ=0.7 resulted in a more pronounced decline to 71.80%. This suggests that while the method tolerates reasonable adjustments to the penalty weight, an excessively large κ makes the policy overly conservative and degrades performance.

**Figure 11:**
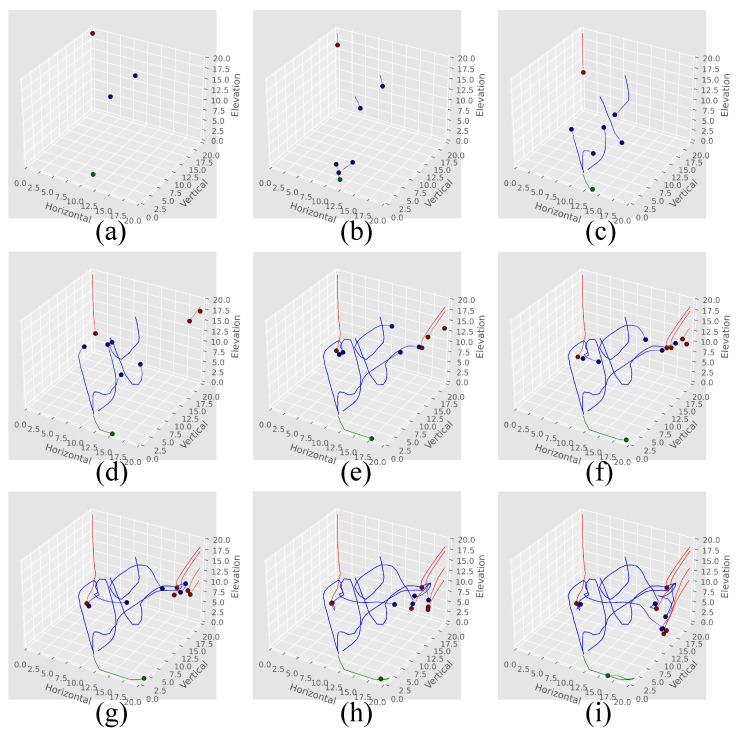
Snapshots of the TAD game with rule-based attackers (5D_vs_5A). (**a**) *t* = 0, (**b**) *t* = 3, (**c**) *t* = 10, (**d**) *t* = 15, (**e**) *t* = 22, (**f**) *t* = 25, (**g**) *t* = 28, (**h**) *t* = 32, (**i**) *t* = 38.

Figure 11 qualitatively illustrates the key emergent intelligent behaviors of the HH-MAPPO framework, providing intuitive validation of its capability to address the core challenges of cooperative defense. Specifically, Figure 11a,b show the upper-level network of the GMT dynamically generates defenders based on the threat situation, demonstrating intelligent resource scheduling. In Figure 11c, the defender team exhibits clear role differentiation (some returning for defense, others continuing interception), which is directly driven by the Role-Aware Embedding (RAE) mechanism, thereby avoiding policy homogenization. Figure 11d,e show that, when confronted with a sudden new attacker, the system can rapidly reassign tasks, transitioning a nearby guard role to interception, proving the real-time coordination capability of the upper-level policy; the subsequent coordinated interception reflects the effectiveness of the lower-level control policy in executing assigned tasks. These behaviors collectively confirm that HH-MAPPO, through its learnable upper-level policy and lower-level control, achieves efficient hierarchical cooperative defense in dynamic environments.

### 4.5. Robustness Against Learning-Based Attackers

To further evaluate the robustness of HH-MAPPO against adaptive adversaries, we replace the rule-based attackers (artificial potential field) with MAPPO [40]. We compare HH-MAPPO with the strong baseline Hungarian+HMAPPO. The interception rates are reported in Table 4.

As shown in Table 4, HH-MAPPO consistently outperforms Hungarian+HMAPPO in all four scenarios. Notably, in the three asymmetric scenarios (3D_vs_5A, 3D_vs_7A, 5D_vs_7A), our method achieves substantial improvements of +16.21%, +8.92%, and +5.57%, respectively, while in the symmetric scenario (5D_vs_5A). the gain is modest (+0.60%). This indicates that HH-MAPPO exhibits superior robustness and adaptability when the defender–attacker ratio is unfavorable.

**Figure 12:**
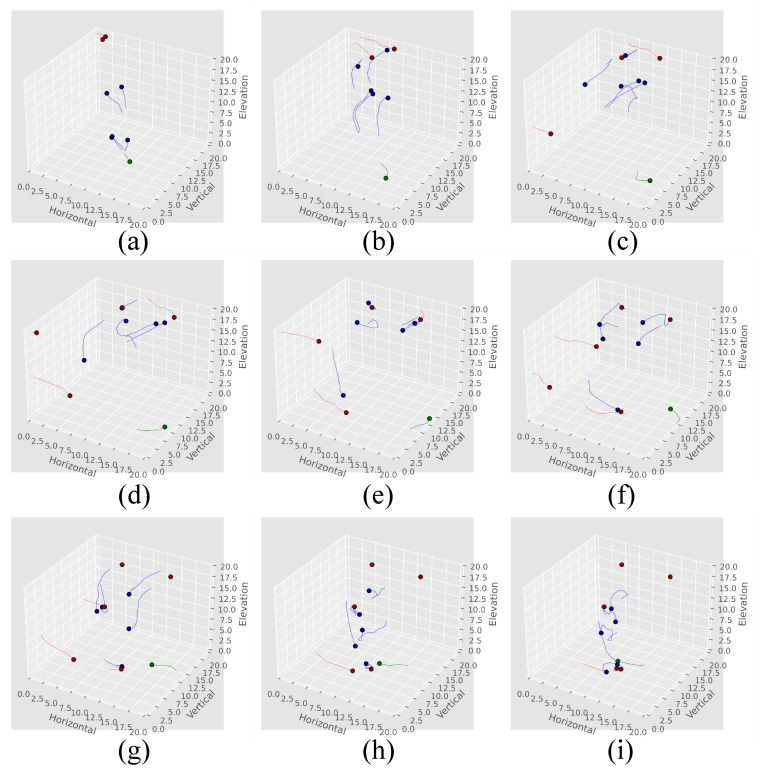
Snapshots of the TAD game with MAPPO-based learning attackers (5D_vs_5A). (**a**) *t* = 4, (**b**) *t* = 14, (**c**) *t* = 20, (**d**) *t* = 25, (**e**) *t* = 32, (**f**) *t* = 38, (**g**) *t* = 44, (**h**) *t* = 51, (**i**) *t* = 55.

Figure 12 provides qualitative snapshots of the confrontation with MAPPO-based attackers. Compared with rule-based attackers, MAPPO-based learning attackers possess stronger environmental adaptability and coordinated penetration capabilities, posing higher challenges to the defense side. Nevertheless, the HH-MAPPO framework still exhibits effective intelligent defense behaviors. Specifically: at moments Figure 12a,b, the defenders concentrate and collaboratively intercept the attackers in the field; at moments Figure 12c,d, the learning attackers launch coordinated penetration, i.e., approaching GMT from multiple directions simultaneously. At this point, the defense side dynamically reassigns tasks through intelligent resource scheduling, reallocating defenders originally responsible for intercepting a certain attacker to more threatening targets. At moments Figure 12e,f, defenders that have completed their interception tasks are reassigned new interception targets, achieving dynamic adjustment of task objectives; at moments Figure 12g–i, facing the continuous penetration of attackers, some defenders actively move forward to intercept, while the remaining defenders move closer to the GMT to protect it.

**Figure 13:**
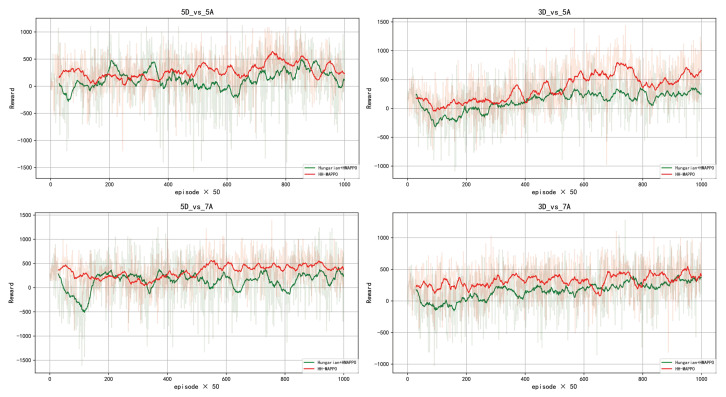
Reward curves of different methods across various scenarios under MAPPO-based attackers.

**Figure 14:**
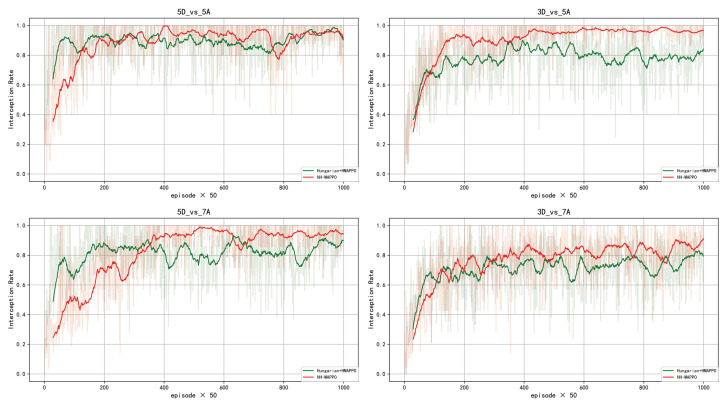
Interception rate curves of different methods across various scenarios under MAPPO-based attackers.

Moreover, the training curves (Figure 13) and interception rate curves (Figure 14) confirm that HH-MAPPO converges faster and maintains a higher final performance.

We compare the results in Table 3 (attackers follow a rule-based artificial potential field) and Table 4 (attackers are governed by a pure MAPPO policy). HH-MAPPO consistently achieves high interception rates against both types of adversaries. Notably, the interception rates are even higher in several scenarios against the MAPPO attackers. This counter-intuitive outcome stems from a fundamental difference in motion control characteristics. A well-tuned potential field produces deterministic, jitter-free trajectories. These trajectories pose an extremely aggressive and immediate threat in high-precision pursuit tasks. In contrast, a reinforcement-learning policy may generate actions with slight oscillations and suboptimality. This is due to exploration noise and the difficulty of convergence in a non-stationary multi-agent setting. Importantly, this observation does not imply that learning-based attackers are inherently weaker. Rather, it highlights a critical open challenge in adversarial games: ensuring motion control stability. HH-MAPPO performs strongly across these two fundamentally different attacker models. This demonstrates that our hierarchical framework learns robust and transferable cooperative strategies, instead of overfitting to a specific adversary type.

### 4.6. Generalization and Scalability

We further examined the generalization capability of HH-MAPPO across different settings and its computational practicality for deployment.

**Generalization to different scenarios.** For the attacker arrival rate, the policy trained with λ=0.1 achieved 97.82% IR at λ=0.05 and 93.99% IR at λ=0.15, indicating robustness to moderate variations in attack intensity. When transferred to a larger 40×40×40 map without retraining, the IR dropped to 31.28%, confirming that the policy is optimized for the spatial scale of the training environment and requires retraining for substantially different maps. For energy constraints, policies independently trained under each budget (E = 30, 50, 70, 100) demonstrate that HH-MAPPO can learn effective strategies from abundant to extremely limited energy.

**Scalability to dynamic defender counts.** A key design feature of HH-MAPPO is its bounded scalability in terms of defender quantity. The observation space is structured with a predefined maximum defender capacity ($N_{\max}=5$ in our experiments), where slots for non-existent defenders are zero-padded. This design provides operational flexibility: once trained, the same model directly supports any number of defenders up to $N_{\max}$ without architectural changes or retraining. However, it is important to note that deploying the policy with a larger $N_{\max}$ (e.g., 10) would still require retraining with an appropriately expanded observation structure, as the framework does not support open-ended swarm scalability.

**Computational cost.** The framework has 3.01 M parameters. Training for $1\times 10^{7}$ steps takes approximately 55 h on a single NVIDIA RTX 4060 GPU, dominated by environment simulation. For deployment, only the actor networks are used, and the average per-step inference latency is 14.37 ms (single defender: ∼2–3 ms). Peak GPU memory usage during inference is only 0.06 GB, making the framework suitable for edge deployment.

## 5. Conclusions

This paper addresses the heterogeneous multi-agent cooperative defense challenge under the coupled conditions of dynamic scale, energy constraints, and strategy homogeneity by proposing a HH-MAPPO algorithm. The core contribution of this work lies in constructing an integrated framework of learnable decision-making and personalized control, which systematically tackles the aforementioned challenges through a three-layer innovative design. Specifically, as a first step, a learnable upper-level policy network replaces traditional fixed rules, achieving joint dynamic optimization of defender quantity and task assignment. Second, the RAE module, by introducing persistent individualized identifiers into the shared network, effectively fosters diverse role-specific behaviors, fundamentally alleviating the strategy homogeneity problem. Finally, the hierarchical target matching and observation filtering mechanisms ensure input dimension stability and computational efficiency under real-time changes in the number of agents.

Experimental validation demonstrates the significant superiority of this framework. In the symmetric scenario (5D_vs_5A) and the asymmetric scenario (3D_vs_5A, 3D_vs_7A, 5D_vs_7A), the interception success rate significantly outperforms baselines. Furthermore, ablation studies confirm that RAE is the key to enhancing team strategy diversity (with a 15.5% increase in the SBAD metric). Additionally, Pareto analysis reveals the algorithm’s excellent performance–energy trade-off capability, maintaining a high interception rate per unit of energy even under extreme energy constraints.

However, the hierarchical reward mechanism proposed in this work relies on a set of manually defined sub-terms and weighting coefficients. While these are shown to be effective through parameter sensitivity analysis, applying the framework to substantially different task scenarios may require careful reward redesign and hyperparameter tuning.

## Figures and Tables

**Table 1:** Agent parameters.

Agent Type	Defender	Attacker	GMT
Max Speed ($|v^{max}|$)	1.0	0.8	0.5
Max Acceleration ($|a^{max}|$)	1.0	0.8	0.5
Initial Quantity	2	1	1
Max Quantity	5	5/7	1
Initial Energy	100	/	100
Energy Utilization Efficiency (χ)	0.8	/	0.9
Propulsion Efficiency Coefficient (μ)	0.75	/	0.5
Deceleration Coefficient (ρ)	0.1	/	0.08
Minimum Operating Energy ($E^{\min}$)	10	/	5
Capture Radius	1.0	1.5	/

**Table 2:** Hyperparameters of the hierarchical reward mechanism.

Parameter	Description	Default
α,β,κ	Weights for $R_{\mathrm{match}},R_{\mathrm{num}},R_{\mathrm{penalty}}$	0.4, 0.4, 0.2
$\zeta_{c},\zeta_{e},\zeta_{r}$	Penalty coefficients (coverage, excess, repeat)	0.5, 0.3, 0.2
$\omega_{c},\omega_{t},\omega_{d}$	Weights for $S_{\mathrm{cover}},S_{\mathrm{threat}},S_{\mathrm{dist}}$	0.5, 0.3, 0.2
$\tau_{c},\tau_{t},\tau_{e}$	Weights for defender’s $w_{\mathrm{cover}},w_{\mathrm{threat}},w_{\mathrm{energy}}$	0.6, 0.3, 0.1
$\eta_{n},\eta_{m}$	Weights for GMT’s $w_{\mathrm{need}},w_{\mathrm{moderate}}$	0.7, 0.3
$D_{\mathrm{threat}}$	Maximum distance for threat evaluation	10.0
$D_{\mathrm{match}}$	Maximum distance for a valid match	8.0
$R_{\min},R_{\max}$	Clipping bounds for individual rewards	−10, 10

**Table 3:** Overall performance comparison of different methods.

Method	5D_vs_5A		5D_vs_7A		3D_vs_5A		3D_vs_7A	
	*IR* (%)	*TE*	*IR* (%)	*TE*	*IR* (%)	*TE*	*IR* (%)	*TE*
HMAPPO	41.77 ± 4.10	154.77 ± 0.23	38.80 ± 4.80	152.60 ± 0.28	45.57 ± 3.66	115.97 ± 0.11	39.64 ± 4.98	110.44 ± 0.12
Auction+HMAPPO	31.22 ± 3.22	145.52 ± 0.18	28.17 ± 2.19	214.50 ± 0.13	48.69 ± 4.75	172.32 ± 0.05	46.54 ± 1.23	178.71 ± 0.16
Hungarian+HMAPPO	77.76 ± 5.66	222.58 ± 0.46	68.03 ± 4.01	244.07 ± 1.30	65.88 ± 3.54	151.67 ± 0.10	57.83 ± 3.74	146.55 ± 0.07
**HH-MAPPO**	93.64 ± 1.43	259.97 ± 0.77	84.80 ± 2.82	232.52 ± 1.60	79.49 ± 2.02	154.08 ± 0.09	80.75 ± 3.33	150.67 ± 0.04

**Table 4:** Interception rate (%) comparison against MAPPO-based attackers.

Method	3D_vs_5A	3D_vs_7A	5D_vs_5A	5D_vs_7A
Hungarian+HMAPPO	79.76 ± 3.32	77.15 ± 3.05	92.64 ± 4.27	88.00 ± 3.16
HH-MAPPO (Ours)	95.97 ± 0.68	86.07 ± 3.58	93.24 ± 2.28	93.57 ± 1.80

## Data Availability

The raw data supporting the conclusions of this article will be made available by the authors upon request.
